# Frequency and correlates of lifetime suicidal ideation and suicide attempts among consecutively hospitalized youth with anorexia nervosa and bulimia nervosa: results from a retrospective chart review

**DOI:** 10.1186/s40479-023-00216-1

**Published:** 2023-03-31

**Authors:** Sabine Arnold, Christoph U. Correll, Charlotte Jaite

**Affiliations:** 1 Department of Child and Adolescent Psychiatry, Psychosomatic Medicine and Psychotherapy, Charité – Universitaetsmedizin Berlin, corporate member of Freie Universitaet Berlin, Humboldt Universitaet zu Berlin, and Berlin Institute of Health, Berlin, Germany; 2 Department of Psychiatry, The Zucker Hillside Hospital, Northwell Health, Glen Oaks, NY USA; 3 Department of Psychiatry and Molecular Medicine, Zucker School of Medicine at Hofstra/Northwell, Hempstead, NY USA

**Keywords:** Eating disorder, Anorexia nervosa, Bulimia nervosa, Suicidality, Suicidal ideation, Suicide attempt, Youth, Inpatients

## Abstract

**Background:**

Youth with eating disorders (EDs) face an increased risk of a premature suicide death. Precursors of completed suicide are suicidal ideation and suicide attempts, which need to be well understood to prevent suicide. However, epidemiological data on the lifetime prevalence and clinical correlates of suicidal ideation and suicide attempts (i.e., “suicidality”) are lacking for the vulnerable group of inpatient ED youth.

**Methods:**

This retrospective chart review was conducted at a psychiatric child and adolescent inpatient department, covering a 25-year period. Consecutively hospitalized youth with an ICD-10 diagnosis of anorexia nervosa (AN), restricting type (AN-R), binge-purging type (AN-BP), and bulimia nervosa (BN) were included. Data extraction and coding were standardized with trained raters extracting information from patient records according to a procedural manual and using a piloted data extraction template. The lifetime prevalence of suicidal ideation and suicide attempts was calculated for each ED subgroup, and clinical correlates of suicidality were analyzed via multivariable regression analyses.

**Results:**

In the sample of 382 inpatients aged 9–18 years (median age = 15.6, females = 97.1%; AN-R: *n* = 242, BN: *n* = 84, AN-BP: *n* = 56), 30.6% of patients had lifetime suicidal ideation (BN:52.4% ≈ AN-BP:44.6% > AN-R:19.8%, χ^2^(2,382) = 37.2, *p* < 0.001, Φ = 0.31), and 3.4% of patients reported a history of suicide attempts (AN-BP:8.9% ≈ BN:4.8% > AN-R:1.7%, χ^2^(2,382) = 7.9, *p* = 0.019, Φ = 0.14). Independent clinical correlates of suicidality were i) for AN-R a higher number of psychiatric comorbidities (OR = 3.02 [1.90, 4.81], *p* < 0.001), and body weight < 1^st^ BMI percentile at hospital admission (OR = 1.25 [1.07,1.47], *p* = 0.005) (*r*^2^ = 0.20); ii) for AN-BP patients a higher number of psychiatric comorbidities (OR = 3.68 [1.50, 9.04], *p* = 0.004) and history of childhood abuse (OR = 0.16 [0.03, 0.96], *p* = 0.045) (*r*^2^ = 0.36), and iii) for BN patients a higher prevalence of non-suicidal self-injury (NSSI)(OR = 3.06 [1.37, 6.83], *p* = 0.006) (*r*^2^ = 0.13).

**Conclusions:**

About half of youth inpatients with AN-BP and BN had lifetime suicidal ideation, and one-tenth of patients with AN-BP had attempted suicide. Treatment programs need to address specific clinical correlates of suicidality, namely, low body weight, psychiatric comorbidities, history of childhood abuse, and NSSI.

**Trial registration:**

This study was not a clinical trial but a retrospective chart review based on routinely assessed clinical parameters. The study includes data from human participants; however: (1) no intervention and no prospective assignment to interventions were performed, and (2) no evaluation of intervention in participants was accomplished.

## Background

Suicide is among the leading causes of death in patients with eating disorders (EDs) [1, 2]. Typical EDs include anorexia nervosa (AN) and bulimia nervosa (BN) [3, 4]. AN is characterized by self-induced underweight, the fear of weight gain, and body image disturbance while covering a restricting (AN-R) and binge-purging (AN-BP) subtype. In contrast, BN is defined by recurrent binge eating and purging episodes, accompanied by vast concerns about body weight and shape [3, 4]. Compared to the general population, youths and adults with AN are eighteen times more likely to die from suicide, and BN patients are seven times more likely [5]. Notably, youths and young adults with EDs are at increased risk for suicide, with rates up to 15% for AN and 2% for BN [6]. Further, suicide rates were higher among young AN patients hospitalized in a psychiatric than in a medical unit [6].

In order to better understand the relationship between suicidal ideation and suicide attempts in EDs, a staging model incorporating motivation and volition as well as contributing factors can be useful [7]. The model by O’Connor and Kirtley [7] includes the stages of pre-motivation, motivation, and volition of suicidal behavior. The first stage incorporates diathesis and stress factors, while the second stage explains the formation of suicidal ideation by factors such as thwarted belongingness, perceived burdensomeness, lacking social support, and reduced resilience and coping mechanisms. Finally, suicidal behavior, such as attempting suicide, is associated with factors such as exposure to suicide, impulsivity, physical pain sensitivity, and fearlessness of death [7]. In the context of EDs, recent studies have identified a link between ED psychopathology and the factors promoting suicidal ideation and suicidal behavior. Specifically, various ED symptoms can relate to feeling thwarted belongingness and burdensomeness [8]. For example, body dissatisfaction may raise the feeling of being a burden to others [9]. Further, starvation, and purging behaviors, as types of self-harm, may enhance pain tolerance and fearlessness about death and, thereby, be considered a path to actual suicidal behaviors [10–12]. Although suicidal ideation and suicide attempts are clinical precursors to suicide, they have been much less studied than completed suicide [6]. Epidemiological data on the lifetime prevalence of suicidal ideation and suicide attempts are crucial to identifying at-risk people and providing care.

### Lifetime prevalence of suicidal ideation and suicide attempts

According to a recent review, one-fourth to one-third of patients with AN and BN have suicidal ideation [5]. Specifically, in a large nationally representative survey of students in grades 7–12 (*n* = 10,123) who were asked if they had ever seriously considered suicide, 53.0% of adolescents with BN and 31.4% with AN reported suicidal ideation [13]. Further, in an adult outpatient sample in which suicidal ideation was evaluated as part of routine initial assessment, 34% of AN-BP patients (*n* = 62) and 20% of AN-R patients (*n* = 104) affirmed they had suicidal ideation [14]. In a study of adolescent BN outpatients (*n* = 21), suicidal ideation was indicated for 42.9% by a rating of their mothers [15]. In young adult inpatients, suicidal ideation was indicated by 65.4% of AN-BP (*n* = 26) and 35.4% of AN-R (*n* = 48) patients, assessed by semi-structured interviews and verified by previous psychiatric records [16]. In a sample of adolescent inpatients, 47% reported suicidal ideation in a self-assessment inventory (combined group of AN: *n* = 36 + AN, atypical: *n* = 8 + BN: *n* = 3) [17].

Considering suicide attempts, a comprehensive review indicated a lifetime prevalence varying from 25–35% in BN patients and 3–20% in AN patients [1]. A population-based survey reported suicide attempts in 35.1% of adolescents with BN and 8.2% with AN, but individuals were only asked about suicide attempts when they endorsed suicidal ideation (*N* = 10,123, age range = 13–18 years) [18]. Among inpatients, the lifetime prevalence of suicide attempts was 2.0% in adult AN-R patients (*n* = 48), assessed via a semi-structured interview [16] being 9% in youth ED inpatients, reported by self-assessment questionnaire (AN: *n* = 36, AN, atypical: *n* = 8, BN: *n* = 3) [17]. Across previous research, suicidality, defined as suicidal ideation and/or suicide attempts, was more prevalent in the binge-purging EDs BN and AN-BP compared to AN-R [1, 14, 19]. Binge and purging behavior was linked to greater difficulties in emotion regulation [20], associated with deficient coping strategies, and more social problems increasing the risk of suicidal ideation [7]. Further, binge-purging behavior correlates with impulsivity [20], which was related to a higher likelihood of acting on suicidal ideation [7, 10]. Altogether, the lifetime prevalence of suicidal ideation and suicide attempts varied widely across age groups and treatment settings. Also, AN subtypes were often not distinguished. Moreover, prevalence data are lacking for suicidal ideation and suicide attempts for the vulnerable group of youth inpatients evaluating AN-R, AN-BP, and BN subtypes.

### Clinical correlates of suicidality

Understanding specific clinical correlates of suicidality is crucial for the ability to tailor suicide prevention and treatment programs. A previous national representative study found suicide attempts to be associated with a longer duration of illness in adults diagnosed with AN (*n* = 276) [19]. In addition, a significant relationship between suicide attempts and ED duration was found in a mixed sample of AN and BN in- and outpatients (AN-R: *n* = 29, AN-BP: *n* = 4, BN: *n* = 13; mean age = 15.7 ± 1.7) [21]. Further, an earlier ED onset was linked to attempted suicide in adults from the community with a BN diagnosis (*n* = 92) [19]. In AN-R, AN-BP, and BN patients, lower body weight was associated with suicide attempts [14, 19].

Considering psychiatric comorbidities, affective disorders, anxiety disorders, and substance use disorders were the most common predictors of suicidal ideation and attempted suicide across studies with adolescent and adult out- and inpatients with AN and BN [1, 6, 14, 19]. Particularly a higher prevalence of a depressive episode and persistent depressive disorder [19], panic disorder, specific phobia, post-traumatic stress disorder [19], and obsessive–compulsive disorder [6, 14] were associated with suicidality. Additionally, personality disorders confer a higher suicide risk [1]. For instance, in patients with AN, antisocial personality disorder, borderline personality disorder, and schizotypal personality disorder were linked to suicide attempts, while schizotypal personality disorder was only related to suicide attempts in patients with BN [19]. Altogether, several psychiatric diagnoses were associated with suicidality, depending on the ED subtype, age group, and treatment setting. However, specific clinical correlates of suicidality in youth inpatients with AN-R, AN-BP, and BN are still unknown.

The complex relationship between EDs and suicidality can be understood in the context of physiological factors of starvation and malnutrition, personality factors such as impulsivity, as well as family and biographical aspects. A recent review including children with and without a psychiatric diagnosis showed suicide in children to be associated with parental psychopathology, maternal suicide attempts, and exposition to suicide within their family or community [22]. Destructive environmental and shared genetic risk can explain the transmission of suicidality [22], as well as the Werther syndrome, which describes emulating the suicide of a role model [23]. Moreover, in previous studies, suicidality correlated with a history of physical, sexual, and emotional abuse [24]. Adverse childhood experiences often develop into negative mental health outcomes [25]. Specifically, in youth AN and BN out- and inpatient populations, sexual and physical abuse correlated with suicidality [1, 15, 16, 21]. Further, in ED samples, suicidality was related to other types of self-harm, such as non-suicidal self-injury (NSSI) [14, 17, 26]. This pattern can be explained by emotion-regulation deficits and personality factors, such as impulsivity, underlying EDs, NSSI, and suicidality [27–30].

Finally, psychotropic medication prescription to prevent suicidality in psychiatric patients is controversial, particularly in child and youth patients. Psychotropic drugs can provide anti-suicidal properties by affecting neuromodulator systems, such as the serotonergic. German treatment guidelines for suicidality in children and adolescents [31] recommend considering supplementary sedative medication for acute suicidality augmenting psychotherapeutic interventions. In chronic suicidality, psychopharmacological therapy of the underlying disease is recommended, for example, antidepressant medicine for depression. Yet, research is needed on suicidality and psychotropic medication prescription in youth inpatients with AN-R, AN-BP, and BN.

### Aims of the present study

Although differentiated epidemiological knowledge is crucial for suicide prevention, prevalence data and clinical correlates for suicidality are lacking for the group of youth hospitalized for EDs. To address these gaps, this study analyzed the lifetime prevalence of suicidality, differentiating suicidal ideation and suicide attempts, as well as independent clinical correlates for suicidality in a consecutively hospitalized youth inpatient sample, comparing the ED subgroups AN-R, AN-BP, and BN. Based on prior findings, we hypothesized suicidal ideation to be reported more frequently than suicide attempts, with suicidality being more prevalent in binge-purging EDs [1, 14, 19]. Further, also based on prior literature [14, 17, 19, 21, 26], we expected a higher ED chronicity and severity, more psychiatric comorbidities, and NSSI as correlates for suicidality.

## Methods

### Study design and ethics

This study was part of a larger retrospective chart review conducted at the child and adolescent psychiatric inpatient department of the University Hospital Charité in Berlin, Germany [32]. Data were treated according to the EU General Data Protection Regulation and the Data Protection Act of 2017 [33]. The patient data were gathered, deposited, and protected within the context of routine hospital care. Before being used for any research purpose, all data were anonymized. The local Ethics Review Board of the University Hospital Charité approved the retrospective chart review study, providing a waiver of patient/caregiver consent.

### Patient sample

Inclusion criteria were as follows: (1) ED diagnosis of F50.00 AN-R, F50.01 AN-BP, and F50.2 BN according to the International Classification of Diseases (ICD-10) [3], (2) age up to 18 years (collectively called “youth”), (3) inpatient treatment, (4) treatment received between 1990 and 2015. All patients were consecutively admitted to the child and adolescent psychiatric department of the University Hospital Charité in Berlin, Germany. If a patient was admitted multiple times, only the first stay was included. In this study, patients with the ICD-10 [3] diagnoses of F50.1 atypical AN, F50.3 atypical BN, F50.4 overeating associated with other psychological disturbances, F50.8 other EDs, and F50.9 unspecified EDs were excluded. Patients received a multimodal ED-specialized inpatient treatment within the German health care system. Treatment comprised body therapy, nutritional counseling, and psychodynamic or cognitive-behavior psychotherapy in an individual and group setting. In particular, body therapy focused on mindful handling of the body and developing an adequate perception and image of the own body.

### Data extraction and coding process

The chart review data collection followed a standardized procedure: First, data extraction sheets were designed, and four independent raters were trained. After familiarizing the study variables, the procedural protocol, and the data extraction templates, the raters extracted and coded patient records for practice. The rater team compared and discussed the coding, supervised by the study’s principal investigator (CJ). After completing the training and revising the extraction templates, the main data acquisition started. Raters retrieved data from paper-based records, including hospital admission, treatment, and discharge information. The extracted information was coded into major and sub-variables, and numerical variables were calculated. CJ monitored the entire data gathering and coding process, and team meetings were held weekly to discuss questions and reach a consensus whenever needed. Further details on the data extraction and coding procedure can be found in Arnold et al. [32].

### Study variables

Psychiatric diagnoses were made upon hospital admission according to ICD-10 criteria [3] by child and adolescent psychiatrists or psychotherapists. Onset and duration of the ED, body weight in kilograms, and body height in meters at hospital admission and discharge were gathered to compute weight changes and absolute weight in BMI percentiles [34, 35]. Further, the presence of family psychopathology was assessed by clinical interview based on a semi-structured departmental form at hospital admission. Moreover, if all criteria except age were met for a personality disorder diagnosis, personality disorder *traits* were diagnosed. A history of child abuse, suicide, or attempted suicide in the patient’s environment, and psychiatric medication prescriptions during treatment were also recorded.

In the clinical setting, patients’ history of suicidal ideation and suicide attempts was systematically assessed by a licensed child and adolescent psychotherapist or psychiatrist with the clinic’s structured psychiatric history guide as part of the medical history at hospital admission. Primary, the patient self-report, and parent report were considered to evaluate suicidality. The treatment team documented indicators of current or past suicidal ideation or suicide attempts during treatment. Finally, the psychotherapist or psychiatrist evaluated suicidality upon hospital discharge. For the chart review, raters underwent expert training in the concept of suicidality, encompassing definitions, and differentiating factors. Suicide was defined as the act leading to one’s own death intended and performed by oneself [36]. A suicide attempt was defined as an intentional but non-fatal act to take one’s own life [36, 37]. Thinking of one’s death by taking one’s own life was considered suicidal ideation [36, 38]. Suicidality was used as a summary category for suicidal ideation and suicide attempts [39]. Based on multi-informant data, the following variables were binarily coded (present = 1/absent = 0): (1) lifetime prevalence of suicidal ideation, (2) lifetime prevalence of suicide attempts, and (3) lifetime prevalence of suicidality. Given the relevance and clinical standard of mandatory assessment of suicidality upon hospital admission, when the psychotherapist, psychiatrist, or any other treatment team member did not record suicidal ideation or suicide attempts in the patient’s chart, its absence was assumed.

### Data analytic strategy

All data analyses were conducted with SPSS Version 27 [40], and the significance level was set at alpha < 0.05. The sample characteristics and the lifetime prevalence of suicidal ideation, suicide attempts, and suicidality were compared between AN-R, AN-BP, and BN subgroups. For nominal data, χ^2^-tests were calculated, and One-Way Analyses of Variance (ANOVA) were conducted for interval data. For post-hoc-testing, pairwise χ^2^-tests were applied for single χ^2^-tests and Tukey HSD for ANOVAs. Within the AN-R, AN-BP, and BN subgroups, patients were categorized into those with vs. without lifetime suicidality. χ^2^-tests and independent-sample t-tests were calculated to test between-group differences in prevalence data and psychiatric comorbidities. Nonparametric tests were applied for data that did not meet the requirements of parametric statistics, such as normal distribution or equal variance. Specifically, the Kruskal–Wallis-test replaced the ANOVA, and the Mann–Whitney U-test replaced the independent t-tests. To determine between-group effect sizes, Cramer’s phi was calculated for χ^2^-tests. For ANOVAs, *t*-tests, Mann–Whitney-U, and Kruskal–Wallis tests, Cohen’s d was assessed as an effect size measure. All effect sizes were classified according to Cohen: ≤ 0.1 = no effect, 0.2–0.4 = small, 0.5–0.7 = intermediate, ≥0.8 = large [41].

Stepwise forward binary logistic regression models for each ED subgroup were calculated to identify independent clinical correlates of suicidality. Suicidality was the dependent variable (present = 1, absent = 0). As potential independent variables, all factors that significantly differed between the respective ED subgroup with vs. without suicidality were considered. To prevent multicollinearity, all except one potential variable were eliminated if one variable was calculated from other dataset variables or if variables were highly correlated. The percentage variance explained by the independent factors, retained in the final regression model, was reflected by Nagelkerke’s metric with the following effect sizes: small < 0.1, intermediate = 0.1–0.3, large > 0.5 [42]. The initial sample included 415 ED patients, while 33 patients were excluded from all analyses for the following reasons: ICD-10 criteria not met in AN patients due to BMI percentiles ≥ 10 (*n* = 17), regular menstruation being present at hospital admission (*n* = 3), or unavailable information on BMI percentile (*n* = 2) or AN subtype (*n* = 7), or insufficient data to verify inclusion criteria beyond AN status (*n* = 4). Since too few patients were male (*n* = 11) or children (*n* = 2) (using the WHO definition of adolescents as people between the ages of 11–19 years old [43], we were unable to compare the results in males vs. females or children vs. adolescents. Therefore, we conducted a sensitivity analysis excluding males and children to test the robustness of our results in the female youth sample.

## Results

### Sample description

Altogether, 382 youth inpatients were analyzed, 63.4% with AN-R (*n* = 242), 22.0% with BN (*n* = 84), and 14.7% with AN-BP (*n* = 56)(see Table 1). In the predominantly female (97.1%) sample, the patients’ age ranged from 9 to 18 years, with a median of 15.6 years (interquartile range:14.3, 16.7). Compared to BN and AN-BP, patients with AN-R had the earliest ED onset at a median age of 14.0 years and were youngest at hospital admission with a median of 14.9 years. Concerning a history of childhood abuse, the BN and AN-BP groups faced childhood abuse more frequently than the AN-R group. Moreover, patients with BN and AN-BP had significantly more psychiatric comorbidities than patients with AN-R, including affective disorders, personality disorder traits, and substance abuse. Overall, the prevalence of psychiatric medication prescription was highest in AN-BP patients at 42.9%, followed by BN patients at 25.0% and AN-R patients at 23.1%.

**Table 1:** Sample description

	**Total** (*n* = 382)		**AN-R** (*n* = 242)		**AN-BP** *(n* = 56)		**BN** (*n* = 84)		**ANOVA** **Kruskal–Wallis-test** **χ**^**2**^**-test**				**Tukey HSD** **Mann–Whitney-U-test** **Pairwise χ**^**2**^**-test**		
									**df**_**1**_ **df**_**2**_	**F** **H** **χ**^**2**^	**p**	**d** **phi**	**AN-R** **vs** **AN-BP**	**AN-BP** **vs** **BN**	**BN** **vs** **AN-R**
**Demographic variables**															
Sex, *n* (%)	-	-	-	-	-	-	-	-	2	1.7	0.426	0.07	**-**	**-**	**-**
Female	371	(97.1)	233	(96.3)	55	(98.2)	83	(98.8)	-	-	**-**	**-**	**-**	**-**	**-**
Male	11	(2.9)	9	(3.7)	1	(1.8)	1	(1.2)	-	-	**-**	**-**	**-**	**-**	**-**
Age at hospital admission, years, *Mdn* (Q1,Q3)	15.6	(14.3,16.7)	14.9	(13.8,16.3)	16.4	(15.3,17.1)	16.4	(15.5,17.2)	2	58.2	** < 0.001**	0.83	** < 0.001**	0.608	** < 0.001**
Age of ED onset, years, *Mdn* (Q1,Q3)	14.1	(13.2,15.3)	14.0	(12.9,15.0)	14.7	(13.6,15.9)	14.6	(13.5,15.7)	2	17.0	** < 0.001**	0.41	**0.001**	0.501	**0.002**
Duration of illness, months, *M* ± *SD*	13.9	± 12.5	11.6	± 11.0	16.6	± 15.1	19.1	± 13.0	2,367	12.7	** < 0.001**	0.53	**0.015**	0.471	** < 0.001**
Treatment duration, days, *M* ± *SD*	92.0	± 50.9	96.6	± 42.5	100.5	± 50.2	72.8	± 67.3	2,376	7.9	** < 0.001**	0.41	0.853	**0.004**	**0.001**
**Anthropometric variables**, *Mdn* (Q1,Q3)															
At hospital admission															
BMI percentiles	1.0	(1.0,5.0)	1.0	(1.0,1.0)	1.0	(1.0,2.0)	43.5	(19.5,73.5)	2	239.6	** < 0.001**	2.67	0.062	** < 0.001**	** < 0.001**
Kgs < 1. BMI percentile	0.0	(-3.1,0.0)	-1.4	(-4.0,0.0)	0.0	(-3.0,0.0)	0.0	(0.0,0.0)	2	75.2	** < 0.001**	0.99	0.065	** < 0.001**	** < 0.001**
At hospital discharge															
BMI percentiles	11.0	(6.0, 18.0)	10.0	(5.0,16.5)	10.0	(4.0,16.0)	38.8	(17.0,74.3)	2	53.8	** < 0.001**	0.86	0.817	** < 0.001**	** < 0.001**
Weight change in kgs/week	0.5	(0.3,0.7)	0.5	(0.4,0.7)	0.5	(0.3,0.6)	-0.0	(-0.3,0.2)	2	80.4	** < 0.001**	1.09	0.064	** < 0.001**	** < 0.001**
**Intelligence**, *n* (%)															
Above average	139	(36.4)	97	(40.1)	19	(33.9)	23	(27.4)	2	4.5	0.104	0.11	**-**	**-**	**-**
Average	235	(61.5)	142	(58.7)	36	(64.3)	57	(67.9)	2	2.4	0.296	0.08	**-**	**-**	**-**
Below average	8	(2.1)	3	(1.2)	1	(1.8)	4	(4.8)	2	3.8	0.149	0.10	**-**	**-**	**-**
**Comorbid psychiatric diagnoses at hospital admission**															
Average number, *Mdn* (Q1,Q3)	0.0	(0.0,1.0)	0.0	(0.0,1.0)	1.0	(0.0,1.0)	1.0	(0.0,1.0)	2	23.0	** < 0.001**	0.49	**0.001**	0.855	** < 0.001**
At least one, *n* (%)	185	(48.4)	96	(39.7)	34	(60.7)	55	(65.5)	2	20.6	** < 0.001**	0.23	**0.004**	0.566	** < 0.001**
Substance abuse	7	(1.8)	0	(0.0)	3	(5.4)	4	(4.8)	2	13.4	**0.002**	0.18	**0.006**	1.000	**0.004**
Affective disorders, at least one	117	(30.6)	61	(25.2)	22	(39.3)	34	(40.5)	2	9.2	**0.010**	0.16	**0.034**	0.888	**0.008**
Persistent affective disorder	62	(16.2)	26	(10.7)	13	(23.2)	23	(27.4)	2	15.0	**0.001**	0.20	**0.013**	0.581	** < 0.001**
Depressive episode	61	(16.0)	37	(15.3)	12	(21.4)	12	(14.3)	2	1.5	0.471	0.06	**-**	**-**	**-**
Recurrent depressive episode	2	(0.5)	0	(0.0)	1	(1.8)	1	(1.2)	2	3.7	0.157	0.10	**-**	**-**	**-**
Anxiety disorders, at least one	11	(2.9)	8	(3.3)	2	(3.6)	1	(1.2)	2	1.1	0.574	0.05	**-**	**-**	**-**
Phobia	8	(2.1)	6	(2.5)	1	(1.8)	1	(1.2)	2	0.5	0.765	0.04	**-**	**-**	**-**
Panic disorder	4	(1.0)	2	(0.8)	1	(1.8)	1	(1.2)	2	0.4	0.808	0.03	**-**	**-**	**-**
Mixed anxiety and depression	2	(0.5)	1	(0.4)	0	(0.0)	1	(1.2)	2	1.1	0.586	0.05	**-**	**-**	**-**
Generalized anxiety disorder	1	(0.3)	1	(0.4)	0	(0.0)	0	(0.0)	2	0.6	0.748	0.04	**-**	**-**	**-**
Obsessive–compulsive disorder	30	(7.9)	23	(9.5)	5	(8.9)	2	(2.4)	2	4.5	0.107	0.11	**-**	**-**	**-**
Adjustment disorder	7	(1.8)	5	(2.1)	1	(1.8)	1	(1.2)	2	0.3	0.875	0.03	**-**	**-**	**-**
Post-traumatic stress disorder	2	(0.5)	1	(0.4)	1	(1.8)	0	(0.0)	2	2.2	0.331	0.08	**-**	**-**	**-**
Personality disorder traits	48	(12.6)	13	(5.4)	10	(17.9)	25	(29.8)	2	35.4	** < 0.001**	0.31	**0.004**	0.163	** < 0.001**
Borderline	27	(7.1)	3	(1.2)	7	(12.5)	17	(20.2)	2	37.2	** < 0.001**	0.31	** < 0.001**	0.336	** < 0.001**
Combined	7	(1.8)	2	(0.8)	1	(1.8)	4	(4.8)	2	5.3	0.068	0.12	**-**	**-**	**-**
Histrionic	5	(1.3)	3	(1.2)	0	(0.0)	2	(2.4)	2	1.5	0.473	0.06	**-**	**-**	**-**
Anankastic	4	(1.0)	4	(1.7)	0	(0.0)	0	(0.0)	2	2.3	0.311	0.08	**-**	**-**	**-**
Anxious	3	(0.8)	1	(0.4)	1	(1.8)	1	(1.2)	2	1.3	0.515	0.06	**-**	**-**	**-**
Mixed and other	2	(0.5)	0	(0.0)	1	(1.8)	1	(1.2)	2	3.7	0.157	0.10	**-**	**-**	**-**
Disorders with onset in childhood/adolescence	7	(1.8)	4	(1.7)	1	(1.8)	2	(2.4)	2	0.2	0.912	0.02	**-**	**-**	**-**
**Family Psychopathology**, *n* (%)															
Family psychopathology present	241	(63.1)	155	(64.0)	41	(73.2)	45	(53.6)	2	5.8	0.054	0.12	**-**	**-**	**-**
Suicide (attempt) environment	38	(9.9)	24	(9.9)	7	(12.5)	7	(8.3)	2	0.7	0.722	0.04	**-**	**-**	**-**
**History of childhood abuse**, *n* (%)															
History of any childhood abuse	44	(11.5)	15	(6.2)	11	(19.6)	18	(21.4)	2	18.4	** < 0.001**	0.22	**0.001**	0.798	** < 0.001**
Emotional	18	(4.7)	5	(2.1)	4	(7.1)	9	(10.7)	2	11.3	**0.004**	0.17	0.068	0.563	**0.002**
Physical	15	(3.9)	7	(2.9)	5	(8.9)	3	(3.6)	2	4.4	0.109	0.11	**-**	**-**	**-**
Sexual	13	(3.4)	3	(1.2)	4	(7.1)	6	(7.1)	2	9.4	**0.009**	0.20	**0.025**	1.000	**0.011**
**Lifetime history of suicidality**, *n* (%)															
Suicidality	120	(31.4)	49	(20.2)	26	(46.4)	45	(53.6)	2	39.0	** < 0.001**	0.32	** < 0.001**	0.408	** < 0.001**
Suicidal ideation	117	(30.6)	48	(19.8)	25	(44.6)	44	(52.4)	2	37.2	** < 0.001**	0.31	** < 0.001**	0.370	** < 0.001**
Suicide attempt	13	(3.4)	4	(1.7)	5	(8.9)	4	(4.8)	2	7.9	**0.019**	0.14	**0.014**	0.484	0.211
**Lifetime history of nonsuicidal self-injury**															
Average number, *Mdn* (Q1,Q3)	0.0	(0.0,0.0)	0.0	(0.0,0.0)	0.0	(0.0,1.0)	0.0	(0.0,1.0)	2	10.5	**0.005**	0.97	** < 0.001**	0.409	** < 0.001**
At least one, *n* (%)	82	(21.5)	20	(8.3)	22	(39.3)	40	(47.6)	2	69.6	** < 0.001**	0.43	** < 0.001**	0.527	** < 0.001**
**Psychiatric medication prescription during inpatient treatment**															
Average number, *Mdn* (Q1,Q3)	0.0	(0.0,1.0)	0.0	(0.0,0.0)	0.0	(0.0,1.0)	0	(0.0,0.8)	2	73.6	**0.001**	0.30	**0.001**	**0.015**	0.759
At least one, *n* (%)	101	(26.4)	56	(23.1)	24	(42.9)	21	(25.0)	2	9.2	**0.010**	0.16	**0.003**	**0.027**	0.730
Antidepressants	76	(19.9)	36	(14.9)	21	(37.5)	19	(22.6)	2	15.1	**0.001**	0.20	** < 0.001**	0.056	0.103
Antipsychotics	51	(13.4)	32	(13.2)	12	(21.4)	7	(8.3)	2	5.0	0.082	0.11	**-**	**-**	**-**
Anxiolytics	2	(0.5)	2	(0.8)	0	(0.0)	0	(0.0)	2	1.2	0.559	0.06	**-**	**-**	**-**

*AN-BP* anorexia nervosa-binge-purging type, *AN-R* anorexia nervosa-restricting type, *BN* bulimia nervosa; disorders with onset in childhood/adolescence comprise Asperger syndrome, combined disorder of conduct and emotions, conduct disorders, elective mutism, emotional disorders of childhood, reactive attachment disorder, stutter, tics; intelligence: above average = IQ > 115, average = IQ 85–114, below average = IQ 70–84; *Mdn* = median; Q1 = 1^st^ Quartile, Q3 = 3^rd^ Quartile, *SD* = standard deviation; personality disorder traits: since patients were younger than 18 years, personality disorders were diagnosed as personality disorder *traits* if all criteria except age were met; significant *p*-values < 0.05 are bold; please note, that this sample description resembles the one in Arnold et al. [32] since the same sample was analyzed

### Lifetime prevalence of suicidal ideation and suicide attempts

Across ED subgroups, a lifetime history of suicidality was observed in 31.4% of patients, ranging from 53.6% in BN patients, via 46.4% in AN-BP patients, to 20.2% in AN-R patients (see Table 1). Specifically, lifetime suicidal ideation was present in 52.4% of BN patients, 44.6% of AN-BP patients, and 19.8% of AN-R patients. In comparison, suicide was attempted by 3.4% of patients, with 8.9% of the AN-BP patient group, 4.8% of the BN patient group, and 1.7% of the AN-R patient group.

### Clinical correlates of suicidality

For each ED subgroup, univariate clinical correlates for suicidality are displayed in Tables 2, 4, and 5, while Table 3 displays the multivariable independent clinical correlates for suicidality from the logistic regression analyses. In the univariate analyses, the following variables were significantly different between youth with AN-R with vs. without suicidality: duration of illness, kgs < 1^st^ BMI percentile, weight change per week, at least one psychiatric comorbidity, the average number of psychiatric comorbidities, affective disorders, namely depressive episode and persistent affective disorder, anxiety disorders, namely panic disorder, obsessive–compulsive disorder, personality disorder traits, namely histrionic personality disorder traits, at least one NSSI method, the average number of NSSI methods, at least one psychotropic drug prescription, and the average number of psychotropic drug prescriptions, specifically antidepressants (Table 2). To avoid multicollinearity, the following variables with less between-group statistically significant difference were not included in the initial model of the multivariable regression analysis for AN-R, entering instead variables with overlapping information and greater statistical significance: (1) *at least one psychiatric comorbidity* was dropped for *average number of psychiatric comorbidities,* (2) *depressive episode* and *persistent affective disorder* were excluded in favor of *affective disorders*, (3) *panic disorder* was excluded in favor of *anxiety disorders*, (4) *histrionic personality disorder traits* was excluded in favor of *personality disorder traits*, and (5) *at least one NSSI method* was excluded in favor of *average number of NSSI methods.* In the final multivariable regression model, weight below the first BMI percentile at hospital admission and more psychiatric comorbidities revealed as independent correlates of suicidality in AN-R (Table 3).

**Table 2:** Univariate clinical correlates of suicidality in patients with anorexia nervosa, restricting type

**AN-R** (*n* = 242)	**Suicidality + ** (*n* = 49)		**Suicidality-** (*n* = 193)		**df** **z**	***t*-test** ***U*-test** ***χ***^***2***^**-test**	**p**	**d** **phi**
Age of ED onset in years, *Mdn* (Q1,Q3)	13.7	(12.5,14.9)	14.0	(12.9,15.1)	-1.4	4013.0	0.167	0.18
Duration of illness, month, *M* ± *SD*	15.2	± 13.7	10.7	± 10.1	60.4	-2.2	**0.035**	0.42
BMI percentiles at hospital admission, *Mdn* (Q1,Q3)	1.0	(1.0,1.0)	1.0	(1.0,1.0)	-0.6	4570.5	0.577	0.05
Kgs < 1^st^ BMI percentile, *Mdn* (Q1,Q3)	0.0	(-2.3,0.0)	-1.2	(-4.4,0.0)	-3.2	3356.5	**0.001**	0.41
Weight change in kg/week	0.5	(0.3,0.7)	0.6	(0.4,0.7)	-2.1	3774.0	**0.037**	0.27
Psychiatric comorbidities, average number, *Mdn* (Q1,Q3)	1.0	(0.0,1.0)	0.0	(0.0,1.0)	-5.1	2781.5	** < 0.001**	0.60
Psychiatric comorbidities, at least one, *n* (%)	35	(71.4)	61	(31.6)	1	25.9	** < 0.001**	0.33
Affective disorders, at least one, *n* (%)	21	(42.9)	40	(20.7)	1	10.2	**0.001**	0.21
Depressive episode	12	(24.5)	25	(13.0)	1	4.0	**0.045**	0.13
Persistent affective disorder	10	(20.4)	16	(8.3)	1	6.0	**0.014**	0.16
Anxiety disorders, at least one, *n* (%)	5	(10.2)	3	(1.6)	1	6.6	**0.010**	0.19
Phobia	3	(6.1)	3	(1.6)	1	1.7	0.099	0.12
Panic disorder	2	(4.1)	0	(0.0)	1	3.7	**0.040**	0.18
Mixed anxiety and depression	1	(2.0)	0	(0.0)	1	0.6	0.202	0.13
Generalized anxiety disorder	1	(2.0)	0	(0.0)	1	0.6	0.202	0.13
Post-traumatic stress disorder	0	(0.0)	1	(0.5)	1	0.0	1.000	0.03
Obsessive–compulsive disorder	9	(18.4)	14	(7.3)	1	4.4	**0.036**	0.15
Personality disorders traits, *n* (%)	7	(14.3)	6	(3.1)	1	7.5	**0.006**	0.20
Borderline	2	(4.1)	1	(0.5)	1	1.7	0.105	0.13
Anankastic	1	(2.0)	3	(1.6)	1	0.0	1.000	0.02
Histrionic	3	(6.1)	0	(0.0)	1	7.5	**0.008**	0.22
Combined	1	(2.0)	1	(0.5)	1	0.0	0.356	0.07
Anxious	0	(0.0)	1	(0.5)	1	0.0	1.000	0.03
Family psychopathology present	35	(71.4)	120	(62.2)	1	1.5	0.228	0.08
Suicide (attempt) environment, *n* (%)	8	(16.3)	16	(8.3)	1	2.0	0.158	0.11
History of childhood abuse, *n* (%)	3	(6.1)	12	(6.2)	1	0.0	1.000	0.00
Physical abuse	2	(4.1)	5	(2.6)	1	0.0	0.632	0.07
Emotional abuse	0	(0.0)	5	(2.6)	1	0.3	0.586	0.04
Sexual abuse	1	(2.0)	2	(1.0)	1	0.0	0.494	0.04
NSSI, average number, *Mdn* (Q1,Q3)	0	(0.0,0.0)	0	(0.0,0.0)	-4.5	3840.5	** < 0.001**	0.26
NSSI, at least one, *n* (%)	12	(24.5)	8	(4.1)	1	18.7	** < 0.001**	0.30
Psychotropic drugs, average number, *Mdn* (Q1,Q3)	0.0	(0.0,1.0)	0.0	(0.0,0.0)	-2.3	3992.5	**0.022**	0.22
Psychiatric drugs, at least one, *n* (%)	17	(34.7)	39	(20.2)	1	4.6	**0.032**	0.14
Antidepressants	12	(24.5)	24	(12.4)	1	4.5	**0.034**	0.14
Antipsychotics	10	(20.4)	22	(11.4)	1	2.8	0.096	0.11
Anxiolytics	1	(2.0)	1	(0.5)	1	0.0	0.365	0.07

*AN-R* Anorexia nervosa, restricting type, *Mdn* median, *NSSI* lifetime non-suicidal self-injury, *psychotropic drugs* prescription during treatment, Q1 = 1^st^ Quartile, Q3 = 3^rd^ Quartile, *SD* standard deviation, *suicidality* + lifetime history of suicidal ideation and suicide attempts, *suicidality*- no lifetime suicidal ideation and suicide attempts, significant *p*-values < 0.05 are bold

**Table 3:** Logistic regression models of multivariable clinical correlates for suicidality in eating disorder subgroups

	**b**	**SE**	**OR**	**95% CI**	**df**	***P*-value**
**Model for AN-R** (*n* = 242), variables						
Kgs < 1^st^ BMI percentile at hospital admission	0.23	0.08	1.25	[1.07,1.47]	1	**0.005**
Psychiatric comorbidities, average number	1.11	0.24	3.02	[1.90, 4.81]	1	** < 0.001**
**Model for AN-BP** (*n* = 56), variables						
History of childhood abuse	-1.85	0.93	0.16	[0.03, 0.96]	1	**0.045**
Psychiatric comorbidities, average number	1.30	0.46	3.68	[1.50, 9.04]	1	**0.004**
**Model for BN** (*n* = 82), variables						
Non-suicidal self-injury, average number	1.1 2	0.4 1	3.06	[1.37, 6.83]	1	**0.006**

*AN-BP* anorexia nervosa, binge-purging type, *AN-R* anorexia nervosa, restricting type, *b* beta, *BN* bulimia nervosa, *CI* confidence interval, *OR* odds ratio, *SE* standard error, significant *p*-values < 0.05 are bold

In the univariate analyses, the following variables were significantly different between youth with AN-BP with vs. without suicidality: at least one psychiatric comorbidity, the average number of psychiatric comorbidities, history of any childhood abuse, specifically childhood sexual abuse, at least one NSSI method, average number of NSSI, and the average number of psychotropic drug prescriptions, specifically antipsychotics (Table 4). To avoid multicollinearity, the following variables with less between-group statistically significant difference were not included in the initial model of the multivariable regression analysis for AN-BP, entering instead variables with overlapping information and greater statistical significance: (1) *at least one psychiatric comorbidity* was dropped in favor of *average number of psychiatric comorbidities,* (2) *sexual abuse* was dropped in favor of *history of childhood abuse*, (3) *at least one NSSI method* was dropped in favor of *average number of NSSI methods,* and (4)* antipsychotics* was dropped in favor of *average number of psychiatric drugs*. In the final multivariable regression model, a history of childhood abuse and psychiatric comorbidities were identified as independent clinical correlates of suicidality in AN-BP patients (Table 3).

**Table 4:** Univariate clinical correlates of suicidality in patients with anorexia nervosa, binge-purging type

**AN-BP** (*n* = 56)	**Suicidality + ** (*n* = 26)		**Suicidality-** (*n* = 30)		**df** **z**	***t*-test** ***U*-test** ***χ***^***2***^**-test**	**p**	**d** **phi**
Age of ED onset in years, *Mdn* (Q1,Q3)	15.0	(13.7,15.8)	15.0	(13.5,16.3)	-0.6	351.0	0.522	0.17
Duration of illness, month, *M* ± *SD*	15.2	± 11.6	17.8	± 17.8	54	0.6	0.520	0.17
BMI percentiles at admission, *Mdn* (Q1,Q3)	1.0	(1.0,2.3)	1.0	(1.0,1.0)	-1.0	340.5	0.296	0.22
Kgs < 1^st^ BMI percentile, *Mdn* (Q1,Q3)	-0.1	(-3.0,0.0)	0.0	(-4.8,0.0)	-0.3	373.0	0.763	0.08
Weight change in kg/week	0.4	(0.3,0.7)	0.5	(0.3,0.6)	-0.3	358.0	0.749	0.09
Psychiatric comorbidities, average number, *Mdn* (Q1,Q3)	1.0	(0.8,2.0)	0.0	(0.0,1.0)	-3.3	204.0	**0.001**	0.90
Psychiatric comorbidities, at least one, *n* (%)	20	(76.9)	14	(46.7)	1	4.1	**0.042**	0.31
Substance abuse disorder, *n* (%)	2	(7.7)	1	(3.3)	1	0.0	0.592	0.10
Affective disorders, at least one, *n* (%)	14	(53.8)	8	(26.7)	1	3.3	0.071	0.28
Persistent affective disorder	9	(34.6)	4	(13.3)	1	2.4	0.111	0.25
Depressive episode	8	(30.8)	4	(13.3)	1	1.6	0.191	0.21
Recurrent depressive episode	1	(3.8)	0	(0.0)	1	0.0	0.464	0.15
Anxiety disorders, at least one, *n* (%)	1	(3.8)	1	(3.3)	1	0.0	1.000	0.01
Phobia	1	(3.8)	0	(0.0)	1	0.0	0.464	0.15
Panic disorder	0	(0.0)	1	(3.3)	1	0.0	1.000	0.13
Obsessive–compulsive disorder	4	(15.4)	1	(3.3)	1	1.2	0.172	0.21
Post-traumatic stress disorder	1	(3.8)	0	(0.0)	1	0.0	0.464	0.15
Personality disorders traits, *n* (%)	6	(23.1)	4	(13.3)	1	0.4	0.487	0.13
Borderline	4	(15.4)	3	(10.0)	1	0.0	0.693	0.08
Combined	1	(3.8)	0	(0.0)	1	0.0	0.464	0.15
Mixed and other	1	(3.8)	0	(0.0)	1	0.0	0.464	0.13
Anxious	0	(0.0)	1	(3.8)	1	0.0	0.464	0.15
Family psychopathology present, *n* (%)	19	(73.1)	22	(73.3)	1	0.0	1.000	0.00
Suicide (attempt) environment, *n* (%)	3	(11.5)	4	(13.3	1	0.0	1.000	0.03
History of childhood abuse, *n* (%)	9	(34.6)	2	(6.7)	1	5.2	**0.016**	0.04
Physical abuse	3	(11.5)	2	(6.7)	1	0.0	0.700	0.16
Sexual abuse	4	(15.4)	0	(0.0)	1	2.9	**0.041**	0.30
Emotional abuse	3	(11.5)	1	(3.3)	1	0.4	0.328	0.09
NSSI, *Mdn* (Q1,Q3)	0.0	(1.0,1.0)	0.0	(0.0,0.0)	-3.8	228.5	**0.002**	0.76
NSSI, at least one, *n* (%)	16	(61.5)	6	(20.0)	1	8.4	**0.004**	0.42
Psychotropic drugs, average number, *Mdn* (Q1,Q3)	1.0	(0.0,2.0)	0.0	(0.0,1.0)	-2.6	253.0	**0.012**	0.63
Psychiatric drugs, at least one, *n* (%)	15	(57.7)	9	(30.0)	1	3.3	0.069	0.28
Antidepressants	13	(50.0)	8	(26.7)	1	2.3	0.128	0.24
Antipsychotics	10	(38.5)	2	(6.7)	1	6.6	**0.007**	0.39

*AN-BP* Anorexia nervosa-binge-purging type, *Mdn* median, *NSSI* lifetime non-suicidal self-injury, psychotropic drugs: prescription during treatment, Q1 = 1^st^ Quartile, Q3 = 3^rd^ Quartile, *SD* standard deviation, *suicidality* + lifetime history of suicidal ideation and suicide attempts, *suicidality*- no lifetime suicidal ideation and suicide attempts, significant *p*-values < 0.05 are bold

In the univariate analyses, the following variables were significantly different between youth with BN with vs. without suicidality: at least one psychiatric comorbidity, the average number of psychiatric comorbidities, at least one affective disorder, depressive episode, at least one NSSI method, and average number of NSSI (Table 5). To avoid multicollinearity, the following variables with less between-group statistically significant difference were not included in the initial model of the multivariable regression analysis for BN, entering instead variables with overlapping information and greater statistical significance: (1) *at least one psychiatric comorbidity* was dropped in favor of *average number of psychiatric comorbidities,* (2) *depressive episode* was dropped in favor of *affective disorders*, and (3) *at least one NSSI method* was dropped in favor of *average number of NSSI methods.* In the final multivariable regression model, a lifetime history of NSSI emerged as the sole clinical correlate of suicidality in BN patients (Table 3).

**Table 5:** Univariate clinical correlates of suicidality in patients with bulimia nervosa

**BN** (*n* = 84)	**Suicidality + ** (*n* = 45)		**Suicidality-** (*n* = 39)		**df** **z**	***t*-test** ***U*-test** ***χ***^***2***^**-test**	**p**	**d** **phi**
Age of ED onset in years, *Mdn* (Q1,Q3)	15.0	(13.7,16.0)	14.4	(13.4,15.3)	-0.8	613.5	0.425	0.19
Duration of illness, month, *M* ± *SD*	17.0	± 12.2	22.0	± 13.6	73	1.7	0.101	0.39
BMI percentiles at hospital admission, *Mdn* (Q1,Q3)	36.0	(18.3,77.8)	46.0	(22.3,64.8)	-0.5	659.0	0.636	0.11
Kgs < 1^st^ BMI percentile, *Mdn* (Q1,Q3)	0.0	(0.0,0.0)	0.0	(0.0,0.0)	-0.9	688.0	0.394	0.04
Weight change in kg/week	0.0	(-0.3,0.2)	-0.0	(-0.3,0.3)	-0.0	288.0	0.968	0.01
Psychiatric comorbidities, average number, *Mdn* (Q1,Q3)	1.0	(1.0,1.5)	0.0	(0.0)	-3.3	545.0	**0.001**	0.69
Psychiatric comorbidities, at least one, *n* (%)	37	(82.2)	18	(46.2)	1	10.5	**0.001**	0.38
Substance abuse	2	(4.4)	2	(5.1)	1	0.0	1.000	0.02
Affective disorders, at least one, *n* (%)	23	(51.1)	11	(28.2)	1	4.6	**0.033**	0.23
Persistent affective disorder	14	(31.1)	9	(23.1)	1	0.3	0.563	0.09
Depressive episode	10	(22.2)	2	(5.1)	1	3.7	**0.031**	0.02
Recurrent depressive episode	1	(2.2)	0	(0.0)	1	0.0	1.000	0.10
Anxiety disorders, at least one, *n* (%)	1	(2.2)	0	(0.0)	1	0.0	1.000	0.10
Phobia	1	(3.8)	0	(0.0)	1	0.0	0.464	0.12
Panic disorder	0	(0.0)	1	(3.3)	1	0.0	1.000	0.10
Mixed anxiety and depression	1	(2.2)	0	(0.0)	1	0.0	1.000	0.10
Obsessive–compulsive disorder	2	(4.4)	0	(0.0)	1	0.4	0.497	0.15
Personality disorders traits, *n* (%)	16	(35.6)	9	(23.1)	1	1.0	0.313	0.14
Borderline	10	(22.2)	7	(17.9)	1	0.0	0.831	0.05
Combined		3 (6.7)		1 (2.6)	1	0.0	0.153	0.10
Anxious	1	(3.8)	0	(0.0)	1	0.0	0.464	0.12
Mixed and other	0	(0.0)	1	(3.8)	1	0.0	1.000	0.10
Histrionic	2	(4.4)	0	(0.0)	1	0.4	0.539	0.15
Family psychopathology present	25	(55.6)	20	(51.3)	1	0.0	0.695	0.04
Suicide (attempt) environment, *n* (%)	5	(11.1)	2	(5.1)	1	0.4	0.442	0.11
History of childhood abuse, *n* (%)	12	(26.7)	6	(15.4)	1	1.0	0.322	0.14
Emotional abuse	5	(11.1)	4	(10.3)	1	0.0	1.000	0.18
Sexual abuse	4	(8.9)	0	(0.0)	1	0.1	0.681	0.07
Physical abuse	3	(6.7)	0	(0.0)	1	1.1	0.245	0.01
NSSI, average number, *Mdn* (Q1,Q3)	0.0	(1.0,1.0)	0.0	(0.0,1.0)	-2.7	611.0	**0.007**	0.54
NSSI, at least one, *n* (%)	27	(60.0)	13	(33.3)	1	6.0	**0.015**	0.27
Psychotropic drugs, average number, *Mdn* (Q1,Q3)	0.0	(0.0,1.0)	0.0	(0.0)	-1.3	765.5	0.184	0.22
Psychiatric drugs, at least one, *n* (%)	14	(31.1)	7	(17.9)	1	1.3	0.256	0.15
Antidepressants	14	(31.1)	5	(12.8)	1	3.0	0.082	0.22
Antipsychotics	3	(6.7)	4	(10.3)	1	0.0	0.099	0.07

*BN* Bulimia nervosa, *Mdn* median, *NSSI* lifetime non-suicidal self-injury, *psychotropic drugs* prescription during treatment, Q1 = 1^st^ Quartile, Q3 = 3^rd^ Quartile, *SD* standard deviation, *suicidality* + lifetime history of suicidal ideation and suicide attempts, *suicidality*- no lifetime suicidal ideation and suicide attempts, significant *p*-values < 0.05 are bold

In the sensitivity analysis, excluding 13 patients (3.4% of the total sample, 11 males (AN-R: *n* = 9, AN-BP: *n* = 1, BN: *n* = 1) and 2 children (AN-R: *n* = 2)), resulting in a sample size of 369 (AN-R: *n* = 231, AN-BP: *n* = 55, BN: *n* = 83), all variables that were significant in the total sample comparing groups with versus without suicidality were also significant in the modified sample without boys and children.

## Discussion

Although youth with severe EDs treated in a psychiatric inpatient setting is assumed to be at an increased risk for suicidality, epidemiological data were lacking for this particular population. To address this gap, this study of almost 400 youths with EDs assessed the lifetime prevalence of suicidality in general and suicidal ideation and suicide attempts in particular. Furthermore, clinical correlates of lifetime suicidality were analyzed.

### Lifetime prevalence of suicidal ideation and suicide attempts

Altogether, in this study, suicidal ideation was reported more frequently than suicide attempts, with suicidality being more prevalent in binge-purging EDs. Specifically, one-third of youth inpatients with AN-R, AN-BP, and BN reported lifetime suicidality. This prevalence estimate aligns with a recent review, noting suicidality in approximately one-third of AN and BN patients across different age groups and settings [5]. In the current study, certain EDs were associated with higher lifetime suicidality than others: about half of BN and AN-BP patients were suicidal compared to one-fifth of AN-R patients. This pattern can be explained by emotion regulation deficits and impulsivity, which are more present in purging EDs than in AN-R [1, 14, 29, 30]. In particular, one in three youths in this study had a lifetime history of suicidal ideation. This finding is in line with previous research, in which one in four to one in three AN and BN patients reported suicidal ideation [5]. Further, patients with BN and AN-BP reported suicidal ideation more often than patients with AN-R, confirming prior studies [1] and the study hypothesis. Specifically, 53% of BN patients had suicidal ideation, which was the same prevalence Crow et al. [13] found in a youth community sample. Our finding of youth AN-BP inpatients reporting lifetime suicidal ideation of 45% is centered in the range of adult AN-BP out- and inpatients (34% [14], 65% [16]). As in Favaro and Santonastaso’s [14] study of adult AN-R outpatients, the prevalence of suicidal ideation in youth AN-R inpatient sample of this study was 20%. Our findings can be well related to previous studies and extend the research by offering lifetime prevalence data for youth inpatients while reporting separate frequencies for AN-R, AN-BP, and BN patient subgroups.

Altogether, 3% of youth-ED inpatients reported at least one lifetime suicide attempt, with the highest prevalence of 9% in AN-BP, followed by 5% in BN and 2% in AN-R. This finding is consistent with prevalence data ranging from 2% in AN-R adult inpatients [16] to 9% in youth AN and BN inpatients [17]. Considering a prevalence between 3 and 35% for suicide attempts indicated by a review of AN and BN patients from various settings and age groups [1], our findings are at the lower end of that range. In light of 35% of community youth with BN having reported at least one lifetime suicide attempt [18], our much lower lifetime prevalence is inconsistent with the higher illness severity that ED patients would have when they need to be hospitalized. However, we assessed the lifetime risk of suicide attempts, and ED patients in the community sample could have been inpatients in the past. Moreover, older samples would have had a longer time to consider and attempt suicide. Furthermore, suicide risk also depends on other factors, such as socioeconomic status, culture, access to care, and suicide prevention measures at the community and school levels [44–46]. Therefore, the relatively low lifetime prevalence of suicide attempts in our sample might depend more on some of these factors than the inpatient status.

### Clinical correlates of suicidality

This study determined univariate and multivariable clinical correlates for suicidality in AN-R, AN-BP, and BN patient groups. Altogether, and in line with the study hypotheses, the identified univariate clinical correlates of suicidality point toward indicators of greater illness severity. Two clinical factors were significantly associated in univariate analyses with suicidality in each ED subgroup: a higher number of psychiatric comorbidities and a higher prevalence of NSSI. The higher number of psychiatric comorbidities is consistent with prior research on youths with BN [13] and adults with AN or BN [19] from community settings, and the association between NSSI and suicidality has also been reported [47, 48]. The relation between these two clinical factors with suicidality calls for a careful assessment and adequate management of psychiatric comorbidities and NSSI in youths with EDs to improve outcomes and prevent suicide. While we found many univariate clinical correlates of lifetime suicidality in youths with ED, other studies have identified some of these in their respective samples [1, 6, 14, 15, 17, 19, 21, 26]. Specifically, univariate analyses in AN-R patients showed a longer duration of illness to be significantly associated with suicidality, which is in line with the association between suicide attempts and a longer AN duration in an adult community sample [19] as well as in adolescent in- and outpatients [21], suggesting that a longer illness of AN can trigger suicidality. In patients with AN-R, a negative association between body weight and suicidality was identified in univariate analyses, which corresponds with previous findings in adolescent AN and BN outpatients [14] and might be explained by the link between suicidal tendencies and a deficiency of certain vitamins and macronutrients [49, 50], and depressive symptoms [51] associated with underweight. Further, in AN-R, depressive episodes, persistent affective disorders, and antidepressant medication prescriptions were approximately twice as common in suicidal patients, as shown in univariate analyses. Similarly, in BN, a depressive episode was four times more common in patients with suicidality. This association between affective disorders and suicidality is consistent with research in ED samples of different age groups and treatment settings [1, 6], including adolescent AN and BN outpatients [15]. Serotonin dysfunction is associated with depressive disorders, suicidality, and AN and BN [52–54], which might be one factor explaining their co-occurrence.

Moreover, in univariate analyses, anxiety disorders were five times more common in the AN-R-suicidality + group, with panic disorder being particularly related to suicidality. The finding of anxiety linked to suicidality is consistent with prior research in adolescent ED outpatients [15] and community adults [19] and might be explained by contemplating suicide to relieve the distress of prolonged agitation, inner tension, and panic attacks [55, 56]. Further, in line with previous studies [1, 19], univariate analyses in this study identified an association between personality psychopathology and suicidality. Specifically, histrionic personality disorder traits were six times more common in suicidal vs. non-suicidal patients with AN-R. In AN-BP, nearly one-fifth of patients with a history of childhood sexual abuse reported suicidality, whereas none of those without suicidality did. These significant associations between sexual abuse and suicidality identified by univariate analyses in this study are consistent with previous findings in adolescent AN and BN in- and outpatients [15, 21]. In addition, antipsychotic medication prescription was more than five times higher in youth with suicidality, which might point to the attempt at treating agitation, inner tension, and impulsivity off-label or even psychotic symptoms experienced in patients with AN-BP and childhood sexual abuse [57].

In addition to the findings of univariate analyses, multivariable analyses in the AN-R patient group identified body weight below the first BMI percentile and a higher number of psychiatric comorbidities at hospital admission as independent clinical correlates of lifetime suicidality. In AN-BP patients, multivariable analyses identified a history of childhood abuse and a higher number of psychiatric comorbidities as independent clinical correlates of suicidality. In BN patients, only the presence of lifetime NSSI remained significant in the final multivariable regression model. To our knowledge, no other study has attempted to identify independent multivariable clinical correlates of lifetime suicidality in youth comparing AN-R, AN-BP, and BN inpatient subgroups.

Our results indicate that different clinical correlates of lifetime suicidality vary in their strength of association with each ED subgroup. In both AN subgroups, psychiatric comorbidities appeared most relevant as targets for addressing suicidality. In contrast, in BN patients, the presence of NSSI seems to be the most robust marker of suicide risk. While these results require replication, they have relevance for the clinical management of ED patients.

### Limitations and future directions

The results of this study need to be interpreted within its limitations. First, similar to most research on suicidality, our design was retrospective, prohibiting causal interferences about ED psychopathology and suicidality as well as assumptions about predictive risk factors for suicide. Second, this was a chart review study. Therefore, only data recorded as regular clinical care could be analyzed. However, records included semi-structured departmental forms, assuring systematic data capture and a high completion rate. Third, in the clinical setting, psychiatric diagnoses were made according to ICD-10 [3] criteria based on the clinical judgment of a licensed psychotherapist or psychiatrist. However, diagnoses did not rely on standardized assessment methods such as semi-structured interviews, warranting reliability. Fourth, the sample size was reduced when ED subgroups with versus without suicidality were created to examine specific clinical correlates, and subgroups had uneven sample sizes. Statistical power was subsequently decreased, and possibly relevant effects could not be detected. Fifth, we included both girls and boys and children and youth, but we could not compare these subgroups due to a small subsample of males and children. However, a sensitivity analysis excluding males and children showed that the results of the current study were highly robust, with all variables that had been identified as significantly different between the patient groups with and without suicidality remaining statistically significant in the subgroup of youth females. Sixth, although we analyzed the frequency of suicidal ideation and suicide attempts separately, the number of patients with suicide attempts was too small to search for independent clinical correlates for each of these separately. Instead, we analyzed clinical correlates of suicidality as the combined clinical feature of suicidal ideation and suicide attempts. Seventh, we focused on inpatient youth with EDs, likely the most severely ill subgroup. Therefore, it is unclear to what degree results generalize to ambulatory youths with EDs without a history of inpatient care. Eighth, we did not formally determine interrater reliability for the data extraction process in this study that relied on a single coding of each chart. While the absence of interrater reliability testing represents a methodological gap in this study, raters received extensive training and were provided with piloted data abstraction templates and a coding manual, including precise variable definitions and coding rules, and data were extracted from a standardized clinical data entry form, mitigating against the effect of lacking interrater reliability testing on data quality. Nevertheless, it is recommended that in future chart review studies, more than one rater codes a patient record to calculate a statistical measure of interrater reliability, such as Cohen’s Kappa [58]. Finally, perhaps we underestimated the lifetime prevalence of suicidality since we assumed its absence when it was not documented. Nevertheless, despite these limitations, to the best of our knowledge, this is the first study that assessed the lifetime prevalence and clinical correlates of suicidality in hospitalized youths with EDs, considering specific ED subgroups, providing previously unavailable information.

## Conclusions

The high prevalence of suicidal ideation in youth inpatients with EDs, especially BN and AN-BP, calls for raising the awareness of this vulnerable population as being at increased suicide risk. Importantly, suicidal ideation and suicide attempts should be considered and assessed thoroughly in each patient with EDs. To effectively address suicidality in youth with EDs, clinicians should consider co-existing psychiatric disorders, low body weight, childhood abuse, and NSSI. More epidemiological research on suicidality is needed to break the stigma and reduce the unreported cases of suicidal ideation and attempted suicides to offer targeted and improved care and save young lives.

## Data Availability

The dataset generated and analyzed during the current study is not publicly available because of privacy and ethical restrictions due to the high data protection of children and adolescents’ data in psychiatric institutions in Germany. However, they are available from the corresponding author on reasonable request.

## Abbreviations

AN-BP: Anorexia nervosa, binge-purging type
ANOVA: Analysis of variance
AN-R: Anorexia nervosa, restricting type
BN: Bulimia nervosa
EDs: Eating disorders
ICD-10: International Classification of Diseases, 10th edition
ICD-11: International Classification of Diseases, 11th edition
NSSI: Non-suicidal self-injury
